# Supplementary material for: Visuomotor decision-making through multifeature convergence in the larval zebrafish hindbrain
Source: Nat Commun. 2026 Mar 5;17:2024. doi: 10.1038/s41467-026-69633-4 (PMC12963418; doi:10.1038/s41467-026-69633-4)
Supplement: Supplementary file 1 — Supplementary Information [file 41467_2026_69633_MOESM1_ESM.pdf]

SUPPLEMENTARY INFORMATION

Visuomotor decision-making through multifeature convergence in the larval zebrafish hindbrain

AUTHOR LIST

Katja Slangewal[*,1,2,3], Sophie Aimon[1,2], Maxim Q. Capelle[1,2,3], Florian Kämpf[1,4], Heike Naumann[1], Krasimir Slanchev[5], Herwig Baier[5], Armin Bahl[*,1,2,3,6,7]

AFFILIATIONS

1) Department of Biology, University of Konstanz, Germany

2) Centre for the Advanced Study of Collective Behaviour, Konstanz, Germany

3) International Max Planck Research School for Quantitative Behaviour, Ecology and Evolution (IMPRS/QBEE); Max Planck Institute of Animal Behavior, Konstanz, Germany

4) Present address: Medical Research Council Laboratory of Molecular Biology, Cambridge, UK

5) Max Planck Institute for Biological Intelligence, Martinsried, Germany

6) Max Planck Institute of Animal Behavior, Konstanz, Germany

7) Zukunftskolleg, Konstanz, Germany

* Correspondence: katja.slangewal@uni-konstanz.de; armin.bahl@uni-konstanz.de

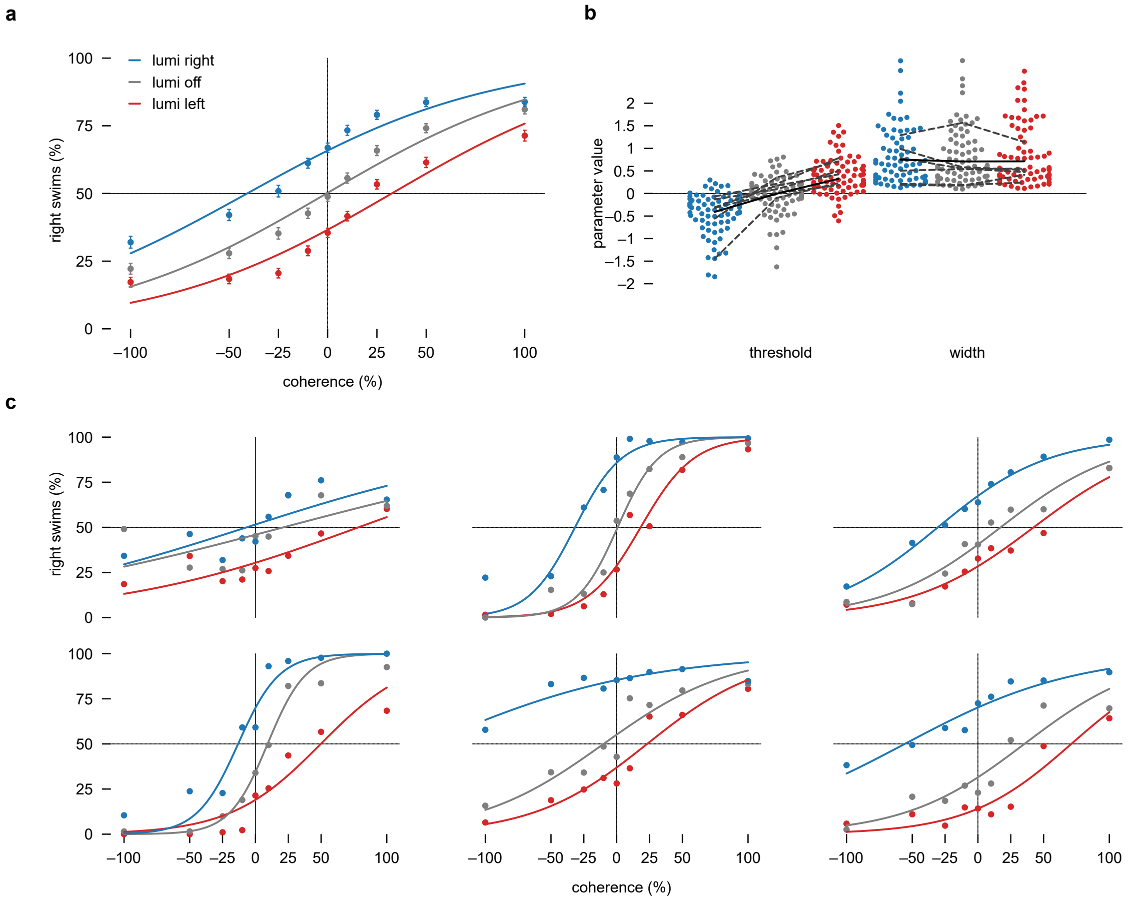

Supplementary Fig. 1 | Fitting of threshold- and width-based psychometric curves reveals changes in threshold rather than width when combining motion and luminance stimuli. (**a**)**,** Same data as in **Fig. 1g,** but fitted with a threshold-and-width-based psychometric curve, also known as the threshold and support parametrization[1,2]. (**b**) Threshold and width parameter estimates for each individual fish (small filled dots). The threshold indicates how much the inflection point is shifted across the x-axis. The width indicates the coding region that contains the 10–90% response rate. Solid black lines are the fit to the mean across all fish. Dashed gray lines belong to the six example fish in (c). (**c**) Six randomly picked example individual fish and their psychometric curve fit. Related to **Fig. 1**.

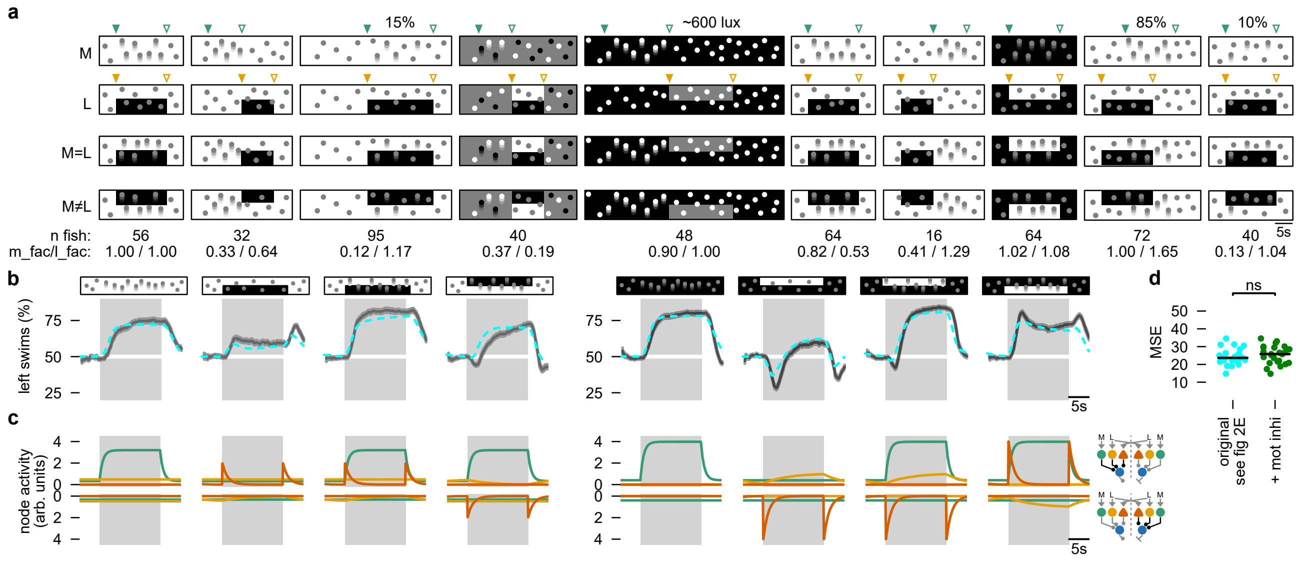

Supplementary Fig. 2 | Stimulus detail overview and model fits. (**a**) Overview of the 10 stimuli used for the behavioral modelling experiments. Green arrowheads (top row) indicate the start (solid) and end (open) of motion. Yellow arrowheads (second row) indicate the start (solid) and stop (open) of the lateral luminance stimulus. Motion coherence was 50% unless indicated otherwise in the top row. White luminance was ~1500 lux, black luminance was ~10 lux, gray luminance was ~900 lux unless indicated otherwise in the top row. The bottom row shows the number of fish per stimulus set, as well as the motion_factor and luminance_factor that were fitted to the data. (**b**) Model fit to the behavioral data shown in **Fig. 2a–d**. Gray lines and shaded curves indicate mean ± SEM. Cyan dashed lines indicate model fit, gray shaded blocks indicate the time when the stimulus was on. (**c**) Simulated contributions of each of the three nodes over time corresponding to the model fits in (b). The upper row contains the contributions to the left multifeature integrator. The bottom row contains the contributions to the right multifeature integrator. Note that the bottom y-axis is mirrored. (**d**) Model fit of the original model, as in **Fig. 2h,** compared to an alternative model with additional inhibition between the left and right motion integrators (green). ns p>=0.05 (two-sided t-test). P-value is 0.440. Related to **Fig. 2**.

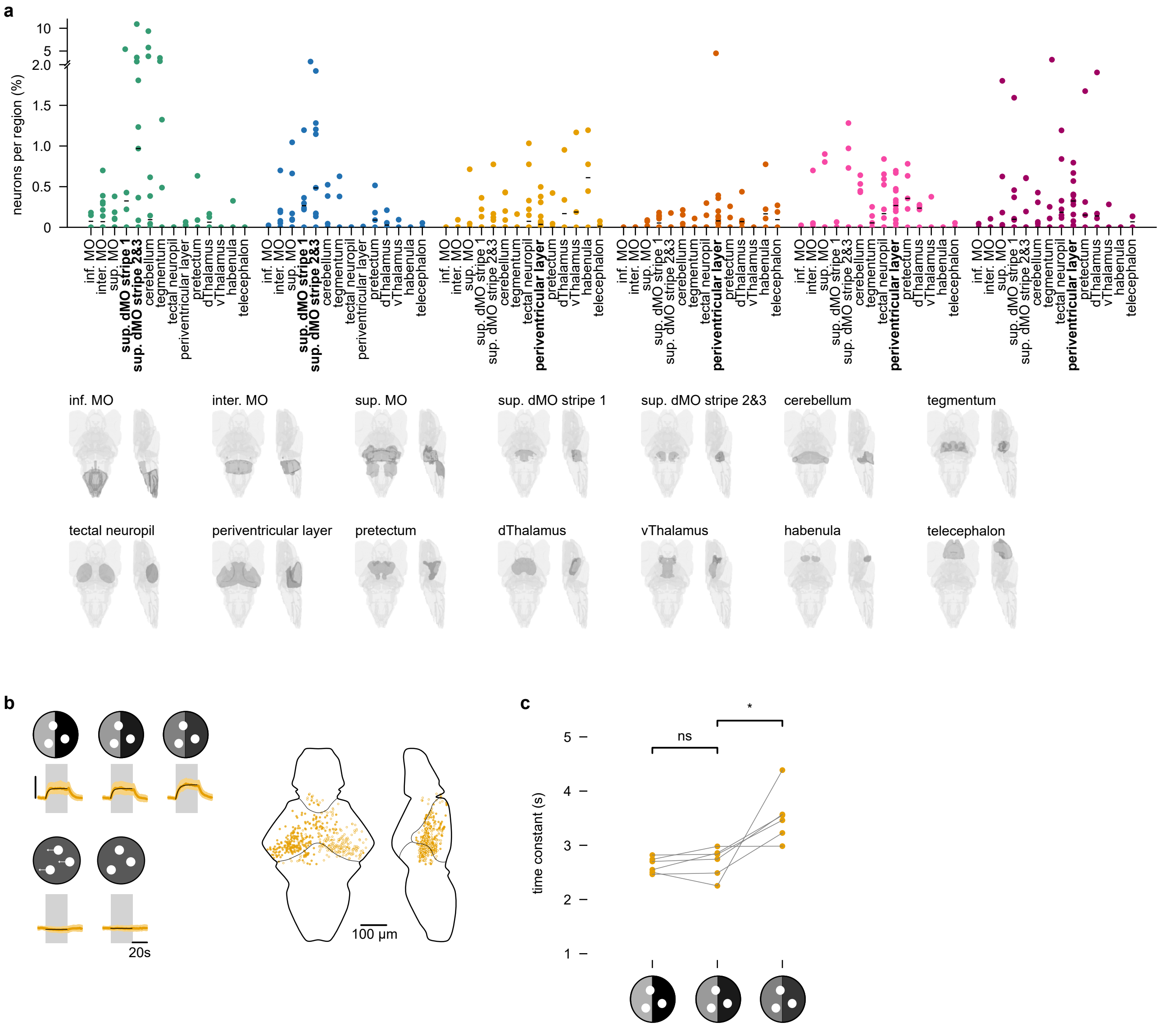

Supplementary Fig. 3 | Overview of functional types across brain regions, corrected for uneven imaging coverage. Temporal integration of luminance integrators. (**a**) Percentage of neuronal cell type per region (as compared to the total number of neurons imaged in that region). Each dot represents a fish. Data is only considered when we imaged at least 100 neurons in the region per fish. Black lines indicate the median percentage. Brain regions are illustrated below. Abbreviations are inf. MO: inferior medulla oblongata, inter. MO: intermediate medulla oblongata, sup. MO: superior medulla oblongata, sup. dMO: superior dorsal medulla oblongata, dThalamus: dorsal thalamus, vThalamus: ventral thalamus. (**b**) Functional activity and location of luminance integrators across three different contrast levels, motion, and during 0 % coherence with luminance off. Vertical scale bar indicates 0.5 dF/F_0. Solid yellow lines and yellow shaded areas indicate median and quartile range. Black curves are fitted exponentials of the median traces: strong contrast time constant: 2.65 s, medium contrast time constant: 2.63 s, and weak contrast time constant: 3.68 s. Gray shaded backgrounds indicate when the stimulus was on. Pre- and post-stimulus consisted of 0% coherence flickering dots on an intermediate gray background. Data for left and right stimulation configurations were merged, as in **Fig. 3f**. In the plot with the map of the brain, filled circles are leftward luminance integrators, open circles are rightward luminance integrators. N=16226 neurons. (**c**) Time constants of the fitted exponential curves to the median dF/F_0 of each fish. The similarity between strong and medium contrast is likely explained by the slow filtering properties of H2B-GCaMP8s. * $p < 0.05$, ns $p>=0.05$ (one-sided t-test; Bonferroni corrected). The p-values are strong vs medium: 0.235, medium vs weak: 0.016. N=6 fish in (b–c). Related to **Fig. 3**.

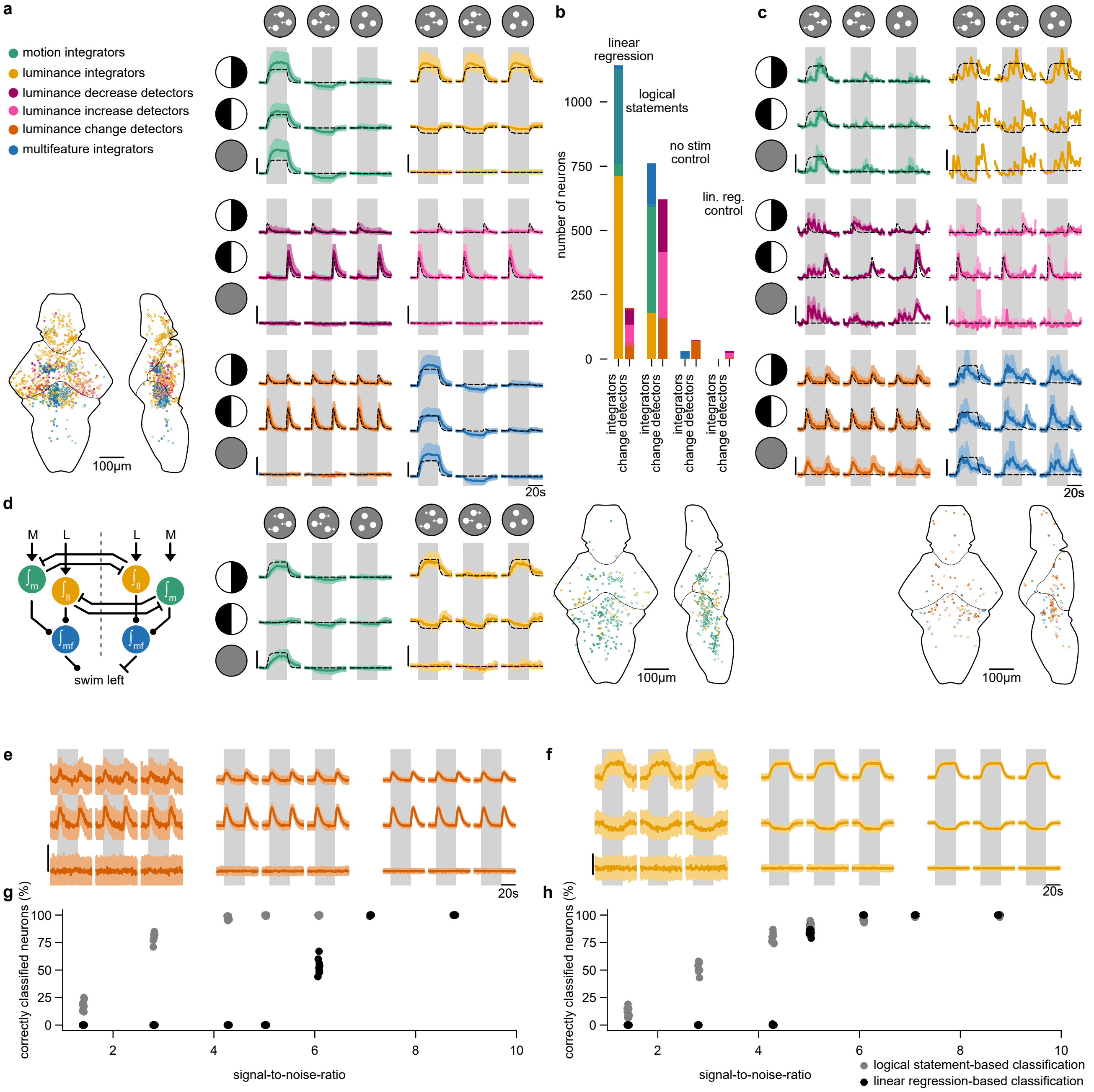

Supplementary Fig. 4 | Linear regression separates functional classes less well than logical statements, control analysis of imaging data reveals no spatial structure, and winner-takes-all representations exist but are spatially distributed. (**a**) Neural activity of neurons found by linear regressor-based correlation to each model node (**Fig. 3e**). (**b**) Number of neurons found by linear regression, logical statements, control analysis using logical statements (no stim control), and control analysis using linear regression (lin. reg. control). Striped blocks indicate neurons that fall into two categories. (**c**) Control analysis, with logical statements applied to randomly selected trials of stimulus *i* (0 % coherence and luminance off). (**d**) Neural activity of neurons found by logical statements matching motion and luminance integrators, who interact through a winner-takes-all mechanism (illustrated in the model cartoon). Colors and brain illustrations in (a,c,d) as in **Fig. 3f,g**. (**e,f**) Synthetic data across 100 simulated luminance change detector neurons (e) and luminance integrator neurons (f) at 3 different signal-to-noise ratios. Synthetic activity consists of the model-based activity, additional Poisson-based random firing, and varying Gaussian noise. The vertical scale bar in the activity examples represents 1 synthetic fluorescence (arb. units). Line and shaded areas are the median response and quartile range of the synthetic data. For comparison, our imaging data has an average signal-to-noise ratio of 5.5. (**g,h**) The correct classification rate across 100 simulated luminance change detector neurons (g) and luminance integrator neurons (h). The percentage of correctly classified neurons by our logical statement approach (gray) and our linear regression approach (black). The performance of the linear regression approach steeply drops for low signal-to-noise ratios; this happens at a higher ratio for the transient luminance change signals, as compared to the constant activity. N=10 sets of 100 simulated neurons. Related to **Fig. 3**.

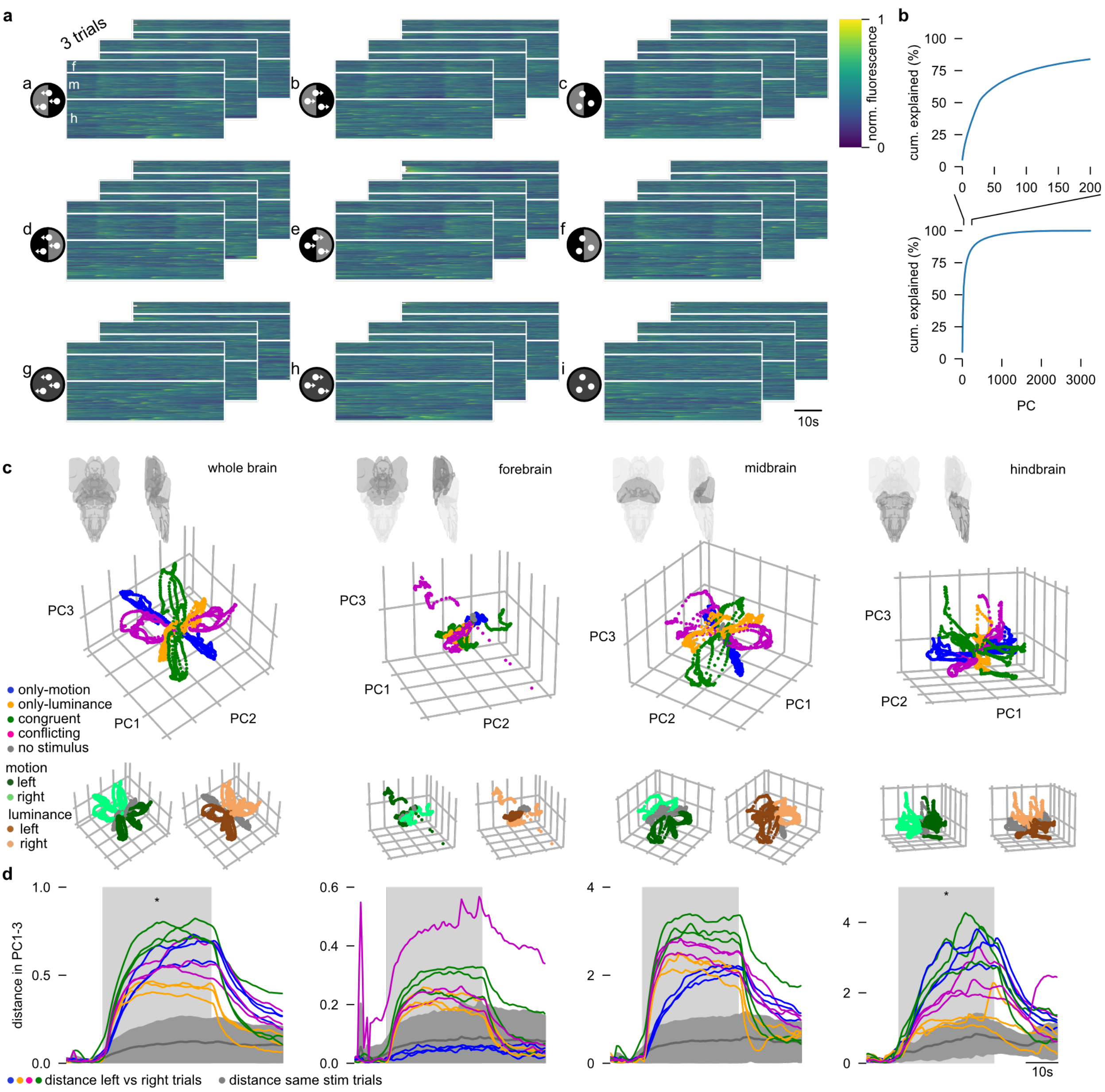

Supplementary Fig. 5 | Unsupervised neural manifold analysis reveals different timescales and separability of congruent and conflicting stimuli across brain regions. (**a**) Overview of the raw data: functional responses of 108118 neurons to 9 stimuli across 3 trials from N=15 fish (same animals as in **Fig. 3**). Traces are ordered by major brain region: f-forebrain, m-midbrain, h-hindbrain. (**b**) Cumulative explained variance across principal components (PCs). Top: zoom-in of the full range in the bottom plot. (**c**) Neural manifold in PCs 1 to 3 for the entire dataset and subsets of the forebrain, midbrain, and hindbrain. The manifold is labeled by stimulus type (only-motion, only-luminance, congruent, conflicting), and below by stimulus direction (left, right). (**d**) Distance in low-dimensional space between two trials of the same type but opposite direction across stimulus time. Gray line and shaded area show the mean ± STD of the distance between 2 trials of the same type in the same direction. Light-gray shaded box indicates the period of stimulus presentation. Stars indicate a significant difference in mean distance over stimulus time (light-gray shaded box) between congruent and conflicting trials according to a two-sided t-test: * $p<0.05$. The p-values are whole-brain: 0.044, forebrain: 0.742, midbrain: 0.067, and hindbrain: 0.028. Related to **Fig. 3**.

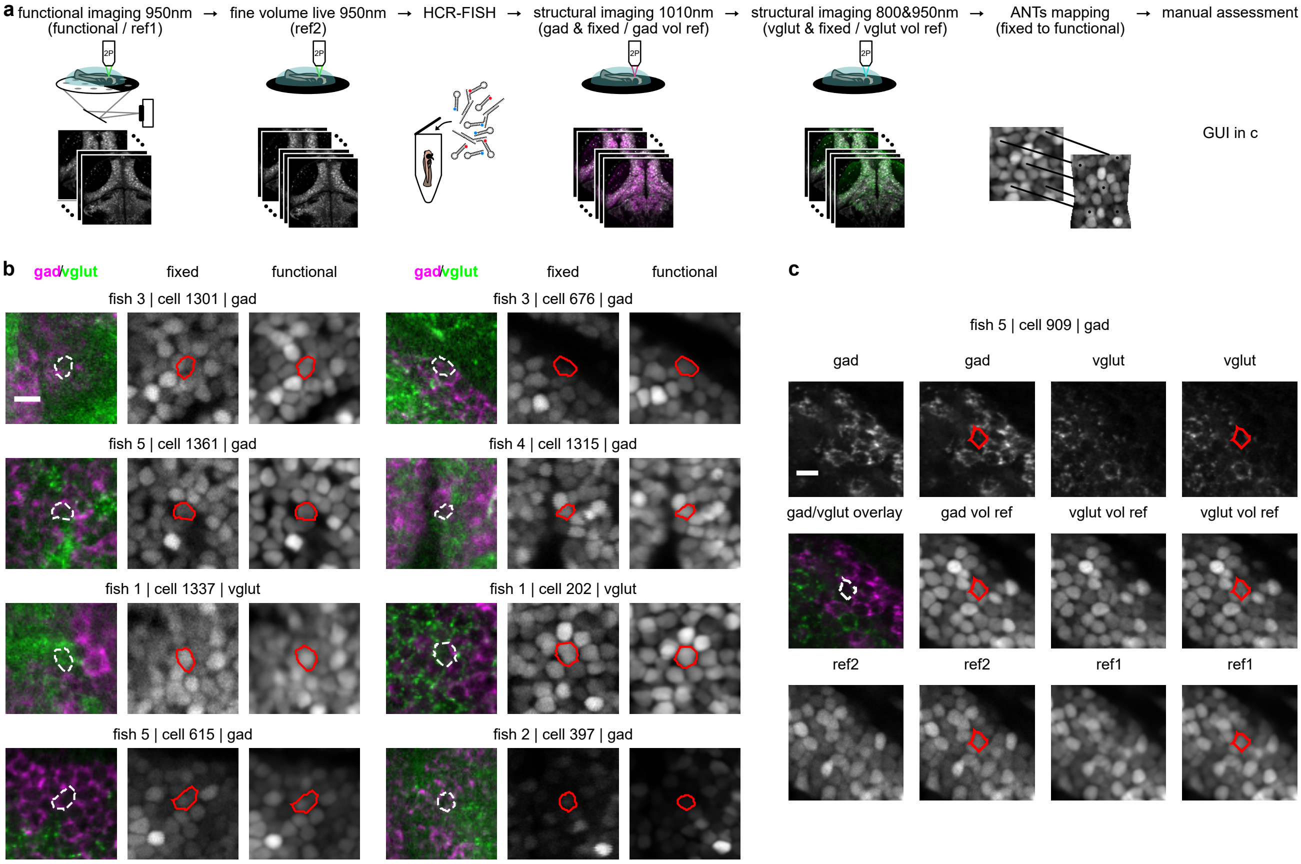

Supplementary Fig. 6 | Classification of cells based on in situ marker expression. (**a**) Schematic of the workflow used to match each neuron's functional type to its molecular identity. Functional imaging (averaged to obtain ref1) was followed by acquisition of a finer structural volume in the live animal (ref2), fixation, and in situ hybridization (HCR-FISH), and high-resolution imaging of the fixed brain. Volumes were aligned using ANTs[3] (fixed to functional reference), and cells were inspected manually in a dedicated GUI. The laser wavelength used for each imaging step is indicated in the title of each step. (**b**) Randomly selected examples of manually classified neurons. These examples are representative of the 315 out of a total of 2240 model-relevant neurons. We assigned high confidence in the neurotransmitter and registration for these 315 neurons. Each example shows a gad/vglut overlay (gad, magenta; vglut, green), the corresponding fixed reference (H2B-GCaMP8s channel), and the functional reference (averaged H2B-GCaMP8s signal). Each cell was labelled as gad-positive or vglut-positive based on the in situ signal. The scale bar is 5 µm. (**c**) Graphical user interface (GUI) used for manual inspection and annotation. The interface displays gad, vglut, and reference (H2B-GCaMP8s) channels for each neuron, enabling verification of marker expression and alignment quality. Cells with clear expression were classified as gad or vglut; those with ambiguous labelling or poor alignment were excluded. The scale bar is 5 µm.

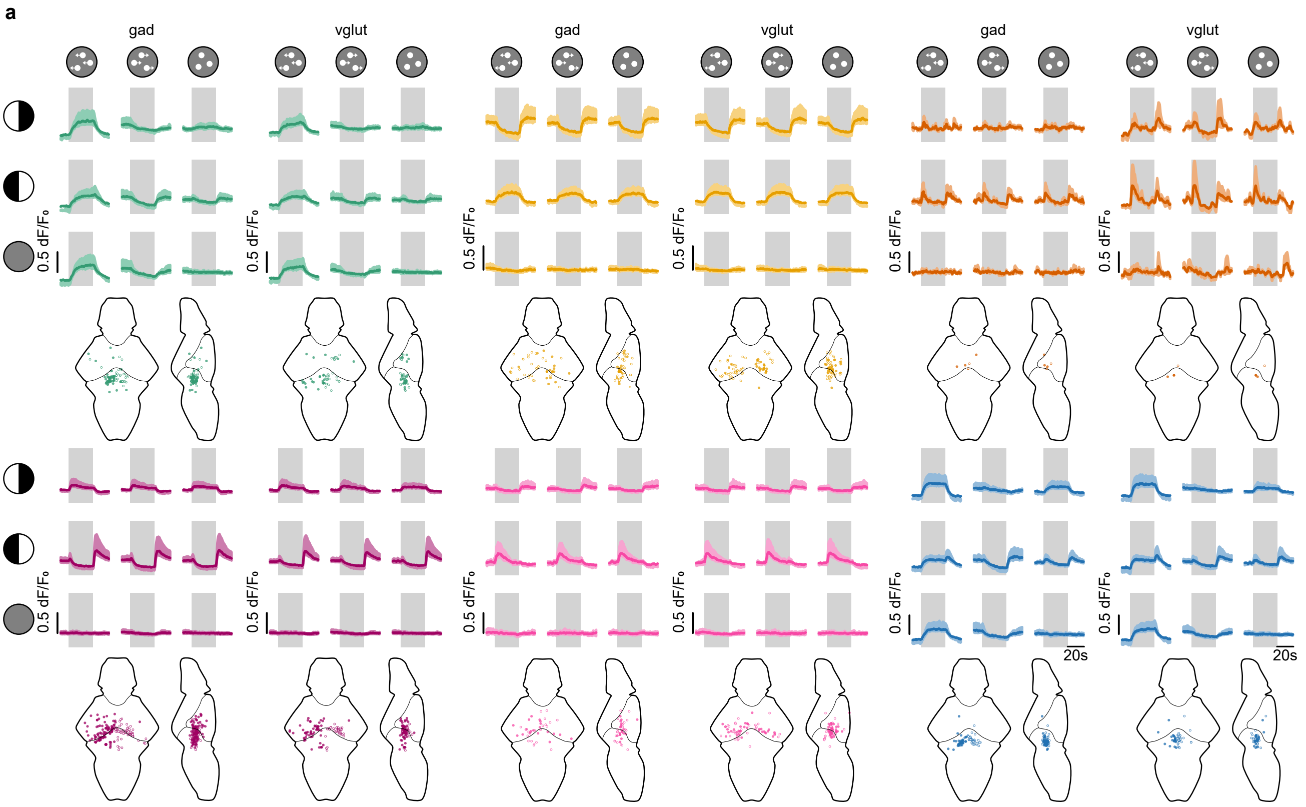

Supplementary Fig. 7 | Gad- and vglut-positive neurons show no clear differences in their functional activity patterns. (**a**) Activity traces and location for each functional cell type split by neurotransmitter type. Left columns: gad; Right columns: vglut. Top row from left to right: motion integrators, luminance integrators, luminance change detectors. Bottom row from left to right: luminance decrease detectors, luminance increase detectors, multifeature integrators. Activity traces and brain maps as in **Fig. 3f**. N=5 fish. Related to **Fig. 4**.

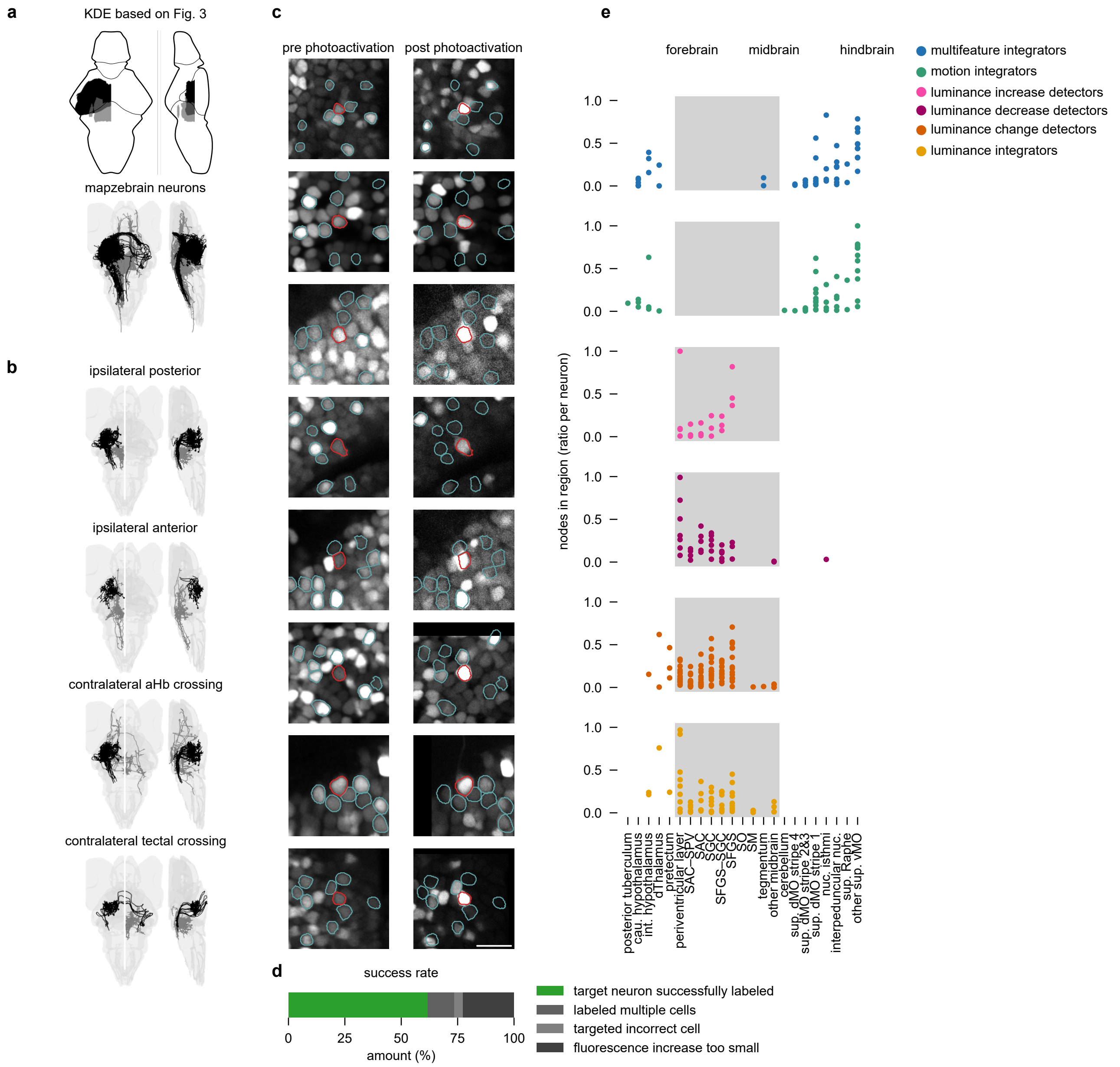

Supplementary Fig. 8 | Projection patterns of neurons through our atlas-based analysis and function-guided photoactivation. Assessment of the precision of the photoactivation method. (**a**) Top: binarized kernel density estimation (KDE) maps, based on data in **Fig. 3**, split into a tectal map (black) and anterior hindbrain map (gray). Bottom: all neurons found in the mapzebrain atlas within each KDE map. (**b**) Selected mapzebrain neurons within the KDE identified regions, split into four potential connection patterns between tectum and anterior hindbrain. (**c**) Confirmation of photoactivating 8 randomly selected target neurons. These examples are representative for the 63 successfully photoactivated neurons. Averaged plane before (left) and after (right) photoactivation. Neurons in the center (red outline) were targeted based on their functional activity. The other outlined neurons (blue) provide visual landmarks to validate mapping results. The outlines for post photoactivation are identical to the ones from pre photoactivation. For volume stacks showing neurites exiting the photoactivated neurons, see **Supplementary Movie 3**. The scale bar is 20 μm. (**d**) Precision of the photoactivation method. Around 60% of our 102 photoactivations were successful, labeling only the single target neuron (green bar). (**e**) Ratio of morphological nodes in a brain region per neuron. Each dot represents one neuron. For clarity, dots at zero are not shown, as cells did not project to such regions. Abbreviations: cau - caudal, int - intermediate, d - dorsal, SAC - stratum album centrale, SGC - stratum griseum centrale, SFCS - stratum fibrosum et griseum superficiale, SO - stratum opticum, SM - stratum marginale, sup - superior, MO - medulla oblongata, nuc - nucleus, v - ventral. Regions are ordered by forebrain, midbrain, and hindbrain. The gray background area distinguishes these three major regions. Related to **Fig. 5**.

SUPPLEMENTARY REFERENCES

1. Kuss, M., Jäkel, F. & Wichmann, F. A. Bayesian inference for psychometric functions. *J. Vis.* **5**, 8 (2005).

2. Houpt, J. W. & Bittner, J. L. Analyzing thresholds and efficiency with hierarchical Bayesian logistic regression. *Vision Res.* **148**, 49–58 (2018).

3. Avants, B. B. *et al.* A reproducible evaluation of ANTs similarity metric performance in brain image registration. *NeuroImage* **54**, 2033–2044 (2011).
