# Supplementary material for: Visuomotor decision-making through multifeature convergence in the larval zebrafish hindbrain
Source: Nat Commun. 2026 Mar 5;17:2024. doi: 10.1038/s41467-026-69633-4 (PMC12963418; doi:10.1038/s41467-026-69633-4)
Supplement: Supplementary file 2 — Description of Additional Supplementary Files [file 41467_2026_69633_MOESM2_ESM.pdf]

Description of Additional Supplementary Files:

Supplementary Movie 1. Behavior examples. One example fish, freely swimming in a 12 cm diameter circular arena. Visual stimulus consisting of coherent dot motion and/or lateral luminance cues. The movie shows in order: motion stimulus, luminance stimulus, congruent stimulus, conflicting stimulus. Each stimulus consists of 10 s pre-stimulus baseline, 30 s stimulus, 10 s poststimulus baseline. The movie is displayed at 2x the original speed. The blue trace was added post-hoc to visualize the recent trajectory of the fish (750 frames; ~8 seconds into the past).

Supplementary Movie 2. Imaging examples. Trial-averaged neural activity of three example imaging planes from three different fish (forebrain - top left; midbrain - bottom left; hindbrain - bottom right). The movie shows in order: motion stimulus, luminance stimulus, congruent stimulus, conflicting stimulus as indicated by the visual stimulus in the top right. Each stimulus consists of 10 s pre-stimulus baseline, 30 s stimulus, 20 s post-stimulus baseline. The neural data movies are displayed at 5x the original speed, the stimulus illustration runs at original speed. Scale bar is 50 µm.

Supplementary Movie 3. Close-up stack postphotoactivation of 8 example neurons. The close-up stack of 8 randomly selected example neurons (matching the neurons in Supplementary Fig. 8c) post photoactivation. A cross indicates the targeted neuron. Arrowheads follow the neurite. Scalebars are 20 micrometer.

Supplementary Movie 4. Photoactivated neurons. Three-dimensional view of the photoactivated neurons in a rotating reference brain. Green: motion integrators, yellow: luminance integrators, pink: luminance increase detectors, purple: luminance decrease detectors, orange: luminance change detectors, blue: multifeature integrators.
