# Supplementary material for: Visuomotor decision-making through multifeature convergence in the larval zebrafish hindbrain
Source: Nat Commun. 2026 Mar 5;17:2024. doi: 10.1038/s41467-026-69633-4 (PMC12963418; doi:10.1038/s41467-026-69633-4)
Supplement: Supplementary file 7 — Reporting Summary [file 41467_2026_69633_MOESM7_ESM.pdf]

Corresponding author(s): Armin Bahl

Last updated by author(s): Jan 12, 2026

# Reporting Summary

Nature Portfolio wishes to improve the reproducibility of the work that we publish. This form provides structure for consistency and transparency in reporting. For further information on Nature Portfolio policies, see our Editorial Policies and the Editorial Policy Checklist.

## Statistics

For all statistical analyses, confirm that the following items are present in the figure legend, table legend, main text, or Methods section.

| n/a | Confirmed | |
|---|---|---|
| ☐ | ☒ | The exact sample size (*n*) for each experimental group/condition, given as a discrete number and unit of measurement |
| ☐ | ☒ | A statement on whether measurements were taken from distinct samples or whether the same sample was measured repeatedly |
| ☐ | ☒ | The statistical test(s) used AND whether they are one- or two-sided<br>*Only common tests should be described solely by name; describe more complex techniques in the Methods section.* |
| ☒ | ☐ | A description of all covariates tested |
| ☐ | ☒ | A description of any assumptions or corrections, such as tests of normality and adjustment for multiple comparisons |
| ☐ | ☒ | A full description of the statistical parameters including central tendency (e.g. means) or other basic estimates (e.g. regression coefficient) AND variation (e.g. standard deviation) or associated estimates of uncertainty (e.g. confidence intervals) |
| ☐ | ☒ | For null hypothesis testing, the test statistic (e.g. *F*, *t*, *r*) with confidence intervals, effect sizes, degrees of freedom and *P* value noted<br>*Give P values as exact values whenever suitable.* |
| ☒ | ☐ | For Bayesian analysis, information on the choice of priors and Markov chain Monte Carlo settings |
| ☒ | ☐ | For hierarchical and complex designs, identification of the appropriate level for tests and full reporting of outcomes |
| ☐ | ☒ | Estimates of effect sizes (e.g. Cohen's *d*, Pearson's *r*), indicating how they were calculated |

*Our web collection on statistics for biologists contains articles on many of the points above.*

## Software and code

Policy information about availability of computer code

| | |
|---|---|
| Data collection | Custom camera tracking and two-photon setup with pre-processing pipeline. See Bahl & Engert, 2020 for detailed description. |
| Data analysis | Custom written Python scripts. Analysis and model code have been archived at the open repository Zenodo and are publicly available at https://doi.org/10.5281/zenodo.18234728. |

For manuscripts utilizing custom algorithms or software that are central to the research but not yet described in published literature, software must be made available to editors and reviewers. We strongly encourage code deposition in a community repository (e.g. GitHub). See the Nature Portfolio guidelines for submitting code & software for further information.

## Data

Policy information about availability of data

All manuscripts must include a data availability statement. This statement should provide the following information, where applicable:

- Accession codes, unique identifiers, or web links for publicly available datasets
- A description of any restrictions on data availability
- For clinical datasets or third party data, please ensure that the statement adheres to our policy

The behavior and preprocessed imaging data reported in this study are publicly available in KonData with the identifier https://doi.org/10.48606/zxctk8418p6jku0t. The preprocessed imaging data contains the raw fluorescent traces for each neuron, as well as the averaged imaging stack. The raw imaging data itself is too large to upload to KonData (~ 3TB). This data will be provided upon request (armin.bahl@uni-konstanz.de) without restrictions. We will respond to requests within 10 working days, and shared data will remain available as long as requested.

## Research involving human participants, their data, or biological material

Policy information about studies with human participants or human data. See also policy information about sex, gender (identity/presentation), and sexual orientation and race, ethnicity and racism.

| | |
|---|---|
| Reporting on sex and gender | No sex or gender can be determined at that age of development. |
| Reporting on race, ethnicity, or other socially relevant groupings | Does not apply for zebrafish. |
| Population characteristics | Does not apply. |
| Recruitment | Does not apply. |
| Ethics oversight | Does not apply. |

Note that full information on the approval of the study protocol must also be provided in the manuscript.

# Field-specific reporting

Please select the one below that is the best fit for your research. If you are not sure, read the appropriate sections before making your selection.

[x] Life sciences   [ ] Behavioural & social sciences   [ ] Ecological, evolutionary & environmental sciences

For a reference copy of the document with all sections, see nature.com/documents/nr-reporting-summary-flat.pdf

# Life sciences study design

All studies must disclose on these points even when the disclosure is negative.

| | |
|---|---|
| Sample size | We based the approximate sample sizes on commonly used numbers across studies (Bahl & Engert, 2020), which have proven successful and sufficient. For behavior this is several tens of fish per experiment. For imaging this is 5-15 fish per experiment. |
| Data exclusions | We used pre-established exclusion criteria to remove tracking-errors, incorrectly photoactivated neurons, and low-confidence HCR labeled neurons.<br><br>For behavior experiments, as mentioned in the methods: “The following bouts were dropped: 1) bouts with an interbout interval > 10 s. This filters out tracking mistakes where the algorithm tracks small particles on the edge of the dish. 2) Bouts where the contour area of the fish exceeded 2000 pixels. This filters out tracking mistakes where accidental air bubbles or scratches got tracked instead of the fish. 3) Bouts with an average speed > 1 cm/s. This filters out tracking mistakes where the algorithm jumps from the edge or bubble back to the fish. 4) Bouts with an orientation change > 150°. This filters out tracking mistakes where the head and tail got swapped 5) Bouts that were within 0.25 cm or less from the edge of the dish. This removal avoided edge effects in our analysis. In addition, we dropped the entire trial, if more than 5% of the bouts were labelled as tracking errors in any of the aforementioned categories. In total, these filtering steps removed 13.7±11.1% of bouts and 14.3±10.1% of trials per experiment.”<br><br>For the photoactivation experiments, as mentioned in Supplementary figure 8: We excluded neurons when neighbouring neurons got co-labeled, when we accidently targeted the wrong functional neuron-type, and when the fluorescence increase was too small to reliably trace the neuron.<br><br>For the HCR-FISH experiments, as mentioned in the methods: “Only neurons that could clearly be identified as GABAergic or glutamatergic with high certainty were included in the analysis. Neurons were excluded if alignment was uncertain, if too few surrounding features were available to assess registration, if the labeling was too noisy, or if gad and vglut signals overlapped too strongly to allow clear discrimination. This approach led us to keep 315 out of the 2240 neurons.” |
| Replication | Experiments have been performed repeatedly across batches and days over 2-3 years. Replication of results is therefore possible. |
| Randomization | Stimuli were given randomly to animals. |
| Blinding | All scripts are automatically analyzing data, no human analysis bias possible. |

# Reporting for specific materials, systems and methods

We require information from authors about some types of materials, experimental systems and methods used in many studies. Here, indicate whether each material, system or method listed is relevant to your study. If you are not sure if a list item applies to your research, read the appropriate section before selecting a response.

## Materials & experimental systems

| n/a | Involved in the study |
|---|---|
| ☒ | ☐ Antibodies |
| ☒ | ☐ Eukaryotic cell lines |
| ☒ | ☐ Palaeontology and archaeology |
| ☐ | ☒ Animals and other organisms |
| ☒ | ☐ Clinical data |
| ☒ | ☐ Dual use research of concern |
| ☒ | ☐ Plants |

## Methods

| n/a | Involved in the study |
|---|---|
| ☒ | ☐ ChIP-seq |
| ☒ | ☐ Flow cytometry |
| ☒ | ☐ MRI-based neuroimaging |

# Animals and other research organisms

Policy information about studies involving animals; ARRIVE guidelines recommended for reporting animal research, and Sex and Gender in Research

| | |
|---|---|
| Laboratory animals | Zebrafish (Danio rerio)<br><br>Wild Type AB<br>Tg(elavl3:H2B-GCaMP8s)mpn438: generated in this study.<br>Tg(alpha-tub:c3pa-GFP)a7437Tg<br>Tg(elavl3:Hsa.H2B-GCaMP6s)jf5Tg<br><br>The three transgenic lines are in a mixed AB and TL genetic background.<br><br>All experiments were done at 5 days post fertilization (dpf). |
| Wild animals | None |
| Reporting on sex | Cannot be determined at 5 dpf. |
| Field-collected samples | Does not apply |
| Ethics oversight | All experiments approved by local Konstanz authorities and the Regierungspräsidium Freiburg, Germany. |

Note that full information on the approval of the study protocol must also be provided in the manuscript.

# Plants

| | |
|---|---|
| Seed stocks | None |
| Novel plant genotypes | None |
| Authentication | None |
